# Recurrent *Staphylococcus warnerii *prosthetic valve endocarditis: A case report and review

**DOI:** 10.1186/1476-0711-10-14

**Published:** 2011-04-23

**Authors:** Ferhat Arslan, Nese Saltoglu, Birgül Mete, Ali Mert

**Affiliations:** 1 Department of Infectious Diseases and Clinical Microbiology, Cerrahpasa Medical Faculty, Istanbul University, Turkey

**Keywords:** *Staphylococcus warneri*, endocarditis, prosthetic valve

## Abstract

To our knowledge, there have been only six *S. warneri *endocarditis cases reported in the English-language literature (Medline: 1966 to April 2011). We report a case of recurrent *S. warneri *endocarditis in a patient with prosthetic valve and silicon mammoplasty and we also review the relevant literature.

## Introduction

Coagulase-negative staphylococci (CoNS) have emerged in recent years as important nosocomial pathogens, with *Staphylococcus epidermidis *the most frequently recognized organism in this group [1]. *S. warneri *and other coagulase-negative staphylococci have been recognized less frequently as significant human pathogens [2].

*S. warnerii *can cause catheter-related bacteremia, native and prosthetic valve endocarditis (PVE), hematogenous vertebral osteomyelitis and ventriculoperitoneal shunt-associated meningitis [3-10]. *S. warnerii *is a CoNS that has rarely been reported in infective endocarditis. To our knowledge, there have been only six *S. warneri *endocarditis cases reported in the English-language literature (Medline: 1966 to April 2011). We report a case of recurrent *S. warneri *endocarditis in a patient with prosthetic valve and silicon mammoplasty and we also review the relevant literature.

## Case Report

A 43-year-old immunocompetent woman presented with a 20-day history of fever and night sweats. On the physical examination, she had an axillary temperature of 38.5°C, a heart rate of 90/min, a blood pressure of 90/70 mmHg and a diastolic murmur (2/4) at the aortic area. Routine chemistry panel and haemogram were normal. The C-reactive protein (CRP) level was 28 g/dl (normal <5) and had an erythrocyte sedimentation rate (ESR) of 30 mm/hour. Three years ago, she had an aortic valve replacement for congenital aortic stenosis. She had a silicon mammoplasty and dental extraction nine months and four months ago respectively prior to this presentation. Three months ago, she had been treated for *S. warneri *endocarditis with ceftriaxone and gentamicin for four weeks. After one month of being discharged from the cardiology department, the patient developed fever and night sweats again. On the second day of last hospitalization, painful nodules (Oslers nodes) were noted in the pad of the right index finger. We initiated empirical infective endocarditis treatment with ampicillin-sulbactam and gentamicin. Transeosphageal echocardiography revealed motile vegetation (15 mm × 4 mm in size) on the non-coronary cusp of the prosthetic aortic valve with no significant aortic incompetence. Three out of six blood culture bottles grew gram positive cocci in clusters, later identified as methicillin-sensitive (CoNS) (subsequently identified as *S. warneri*). We used API ID 32 Staph system (a commercial test system strip consists of 32 cupules, 26 of which contain dehydrated biochemical media for colorimetric tests to identify staphylococci) (Bio-Merieux) for the definitive identification of this bacterium.

On the seventh day of the treatment for the second attack, she developed right hemiparesis and aphasia. Computed tomography analysis showed a hypodense lesion in the left fronto-temporal area compatible with infarction and cerebral angiography revealed an aneurysm at the bifurcation of the middle cerebral artery. The aneurysm was successfully obliterated by a neurosurgical intervention and hemorrhagic lesion eventually resolved. Ampicillin-sulbactam and gentamicin was continued for a total of eight weeks. She survived with minimal sequelae.

## Discussion

PVE is usually health care-related and the infection occurs in 1% to 3% of patients in the first postoperative year of valve surgery. PVE is caused by CoNS in approximately 15-40% of the cases. *S. epidermidis *is the most common organism associated with prosthetic valve endocarditis [11-13]. Especially, *S. lugdunensis, S. capitis *have several virulence factors and have been described to cause destructive native and prosthetic valve endocarditis [14-16]. *S. warneri *rarely causes septicemia and endocarditis. The case presented here is the seventh endocarditis case due to *S. warneri *reported in the literature [4-7,9,10]. The relevant clinical data regarding our case and the patients with *S. warneri *endocarditis reported in the literature are given in Table 1. The mean age of patients was 57 and all except three had predisposing disease. Aortic valve was involved in six of the patients and two patients had concomitant mitral valve involvement. One patient had only mitral involvement. Vegetation was observed in all of the patients on ecocardiography. The mean duration of treatment was 6 weeks (4-8 weeks) and the beta-lactam and aminoglycoside combination was the preferred treatment choice. Three of the isolates were methicillin resistant but vancomycin sensitive. Ganesh D et al. were not reported their isolate sensitivity and the patient treated with nafcillin for 6 weeks [10].

**Table 1:** Clinical features and prognosis of patients with *Staphylococcus warneri *endocarditis reported in the literature

Reference	Age/Gender	Predisposing condition	Valve involved	Presence of vegetation on echocardiography	Antibiotics and duration of therapy
4(1984)	32/M	none	Aortic valve	Yes	Penicilin+ Gentamicin (4 weeks)
6(1989)	66/M	disc prosthesis**	Mitral, Aortic Valve	yes	NA*
3(1992)	64/M	none	Mitral, Aortic Pulmonary Valve	No	Vancomicin+ Gentamicin
7(2001)	71/M	Prosthetic aortic valve	Prosthetic aortic valve	yes	Vancomicin+ pefloxacine (6 weeks)
9(2006)	48/M	disc prosthesis**	Aortic valve	yes	Vancomicin+Fucidic acid (three day) Then Rifampicin+Fucidic acid
10(2011)	78/W	none	Mitral valve	yes	Nafcicillin (6 weeks)
PRESENT CASE(2008)	43/W	Prosthetic aortic valve, silicon mamoplasty**	Prosthetic aortic valve	yes	Ampicillin/sulbactam+ Gentamicin 2 weeks, Then 4 weeks amp/sulb. Ampicilin/sulbactam ampicillin/sulbactam (8 weeks) Not done

A case with infective endocarditis (a 71-y-old male with a history of severe rheumatic aortic stricture, and replacement of the aortic valve by a mechanic prosthetic valve due to *S. warneri *has been reported by Abgrall S al[7]. That patient was afebrile, and three blood cultures were negative. The patient was empirically treated intravenously with vancomycin and gentamicin. Twenty days later the patient went to the aortic valve replacement for increased transvalvular pressure gradient. Upon surgery, two vegetation tags and the valve revealed *S. warneri *on culture. In our case; *S. warneri *was isolated from blood during both the first and the second infective endocarditis attack.

The increasing use of prosthetic devices and intravascular catheters in contemporary healthcare has increased the importance of CoNS as a cause of prosthetic valve endocarditis. CoNS can colonize and produce a biofilm on a biomaterial. This is an important virulence factor in causing foreign body infections which are more indolent by nature. There are previously reported two cases of *S. warneri *endocarditis in which prosthesis (disc prosthesis) were thought to be the resource of infection [6,9]. Endocarditis took place nine months and one year after implantation, respectively. Our patient had silicon mammoplasty six months before the first attack. *S. warneri *endocarditis may directly develop due to procedures with foreign bodies for the heart such as prosthetic mechanic valve replacement, intracardiac defibrillator and pacemaker implantation besides other procedures with foreign bodies such as disk prosthesis. We thougt that *S. warneri *endocarditis may be associated with a catheter or other foreign materials such as silicon prosthesis/mechanic valve in the nosocomial setting.

As a conclusion; *S warneri *infective endocarditis is an uncommon cause of infective endocarditis. This case report is important because of the recurrence of *S. warneri *endocarditis. The reason for recurrence of *S. warneri *endocarditis may be attributed to the biofilm occurrence on the foreign material and shortness of the treatment duration. The effective treatment should be maintained for at least 6-8 weeks for endocarditis due to *S. warneri*.

## Competing interests

The authors declare that they have no competing interests.

## Consent

Written informed consent was obtained from the patient for publication of this case report and accompanying images. A copy of the written consent is available for review by the Editor-in-Chief of this journal.

## Authors' contributions

BM carried out the microbiological study, NS and FA followed the patient' s clinical condition and wrote the paper. AM designed the study. All authors read and approved the final manuscript.
